# Shared decision making in surgery: a scoping review of patient and surgeon preferences

**DOI:** 10.1186/s12911-020-01211-0

**Published:** 2020-08-12

**Authors:** Laura A. Shinkunas, Caleb J. Klipowicz, Erica M. Carlisle

**Affiliations:** 1 Program in Bioethics and Humanities, University of Iowa Carver College of Medicine, Iowa City, USA; 2 Department of Anthropology, University of Iowa, Iowa City, USA; 3 Department of Surgery, University of Iowa Hospitals and Clinics, Iowa City, USA

**Keywords:** Surgery, Shared decision making, Ethics

## Abstract

**Background:**

Many suggest that shared decision-making (SDM) is the most effective approach to clinical counseling. It is unclear if this applies to surgical decision-making-especially regarding urgent, highly-morbid operations. In this scoping review, we identify articles that address patient and surgeon preferences toward SDM in surgery.

**Methods:**

We used the Preferred Reporting Items for Systematic Reviews and Meta-analyses extension for Scoping Reviews (PRISMA-ScR) to develop our protocol. Medline, EMBASE, and Cochrane databases were searched from inception through 11.2017. Title/abstract review identified peer-reviewed, empirical articles that addressed patient/surgeon preferences toward SDM in surgery. Identified articles underwent full review by two independent investigators. We addressed the following questions: (1) What is known from existing empirical evidence about patients’ and/or surgeons’ surgical decision-making preferences? (2) Why might patients and/or surgeons prefer SDM? (3) Does acuity of intervention impact surgical decision-making preferences? Outcome measures included study methods, surgical specialty, diagnosis, study location/setting, type/number of subjects, acuity of intervention, surgeon/patient decision-making preferences, and factors associated with favoring SDM. Data was analyzed in Microsoft Excel.

**Results:**

20,359 articles were identified with 4988 duplicates, yielding 15,371 articles for title/abstract review. 74 articles were included in final analysis. 68% of articles discussed oncologic decision-making. 46% of these focused on breast cancer. 92% of articles included patients, 22% included surgeons. 75% of articles found surgeons favored SDM, 25% demonstrated surgeons favored surgeon guidance. 54% of articles demonstrated patients favored SDM, 35% showed patients favored surgeon guidance, 11% showed patients preferred independent decision-making. The most common factors for patients favoring SDM included female gender, higher education, and younger age. For surgeons, the most common factors for favoring SDM included limited evidence for a given treatment plan, multiple treatment options, and impact on patient lifestyle. No articles evaluated decision-making preferences in an emergent setting.

**Conclusions:**

There has been limited evaluation of patient and surgeon preferences toward SDM in surgical decision-making. Generally, patients and surgeons expressed preference toward SDM. None of the articles evaluated decision-making preferences in an emergent setting, so assessment of the impact of acuity on decision-making preferences is limited. Extension of research to complex, emergent clinical settings is needed.

## Background

Over the past several decades, physician paternalism has been systematically rejected and respect for patient autonomy has emerged as a leading ethical priority in clinical counseling [1]. Shared decision-making (SDM), a process by which physicians and patients actively work together to integrate care plans that are responsive to patient goals and values, has been advocated as a clinical counseling approach that promotes patient autonomy by encouraging patients to participate in clinical decision making [1–4]. Along with its presumed promotion of patient autonomy, data suggesting that SDM reduces health care costs and improves quality of care have led to relatively widespread incorporation of SDM into health policy [4]. Despite this implicit acceptance of SDM, relatively limited data exist regarding patient or physician preferences toward SDM. Such data seem to be especially lacking in surgical decision-making.

By supporting patient autonomy, SDM places some limits on the extent to which a physician’s influence guides a patient’s decisions. Some ethicists have argued that such prioritization of patient autonomy is critically important, and that even subtle attempts by a physician to sway a patient toward a particular decision violates respect for patient autonomy [5]. However, others have argued that if attempts to promote patient autonomy are too strong or rigid, the emphasis on self-determination may be inconsistent with patients’ wishes for more professional guidance [6, 7]. In fact, there is an emerging body of literature that suggests that patients may prefer more physician guidance during medical decision making [6, 8–11]. These findings prompt one to question whether autonomy-heavy approaches to SDM in clinical counseling are always consistent with patient preferences or whether patients would (at least sometimes) prefer a less autonomous and more guided approach to clinical counseling.

With respect to the physician’s perspective, it is important to note that studies have shown physicians to be somewhat reluctant to incorporate SDM into clinical practice [12]. One reason for this may be a sense that when a physician overly prioritizes patient autonomy, there is lessening of the physician’s role such that the fiduciary nature of the patient-physician relationship is undermined. Prioritization of patient autonomy and integration of SDM into clinical counseling has left some physicians feeling that their role has become one of merely offering patients the information necessary to make their own “informed” decisions rather than truly engaging in a fiduciary relationship with the patient [7]. This is illustrated in a recent narrative that describes an encounter in which a physician reviewed all options for treatment of nonischemic cardiomyopathy with her patient but was stopped by the patient before she could make a recommendation with the request that the patient be allowed time to independently reflect and make a decision that was best for him. In the physician’s reflection on the encounter, she notes, “since the decision was his, it was no longer mine. I had informed him. But had I been his doctor?” [7]. Perhaps such efforts to assure patient autonomy and SDM limit the role of the physician in patient counseling. These types of reports call for further investigation so we can better understand physician preferences toward shared decision making.

Concerns about the appropriateness of SDM may be particularly pronounced in surgical decision making given the often dramatic and irreversible outcomes associated with surgery. These concerns may further escalate when considering emergent, highly complex operations that are associated with a high risk of mortality or morbidity. In an initial effort to better understand preferences toward SDM in surgical decision making, we reviewed the literature regarding parent and surgeon preferences toward SDM in pediatric surgery [13]. We found that there was markedly limited data available. Of the 36 existing articles, the predominant focus was on parent preferences toward decision making in elective, non-urgent procedures. There was limited data regarding surgeon preferences and virtually no discussion of preferences for decision making in more urgent settings [13].

The purpose of this review is to gain a more thorough understanding of patient and surgeon preferences toward SDM in adult surgery. We chose to conduct a scoping review because there is limited published data on patient and surgeon decision making preferences, particularly when surgery is considered urgent or emergent. Scoping reviews are a valuable methodology because they allow for the mapping of important concepts and research gaps in a defined area of study by comprehensively identifying, reviewing, and summarizing the existing information from the literature [14]. Specific research questions addressed in our scoping review included: (1) What is known from existing empirical evidence about patients’ and/or surgeons’ surgical decision-making preferences? (2) Why might patients and/or surgeons prefer SDM? (3) Does acuity of intervention impact surgical decision-making preferences?

## Methods

### Protocol design

Our scoping review protocol follows Arksey and O’Malley’s methodological framework [14] as well as the Preferred Reporting Items for Systematic Reviews and Meta-analyses extension for Scoping Reviews (PRISMA-ScR) [15]. This protocol has not been registered.

### Identifying relevant studies

**Fig. 1:**
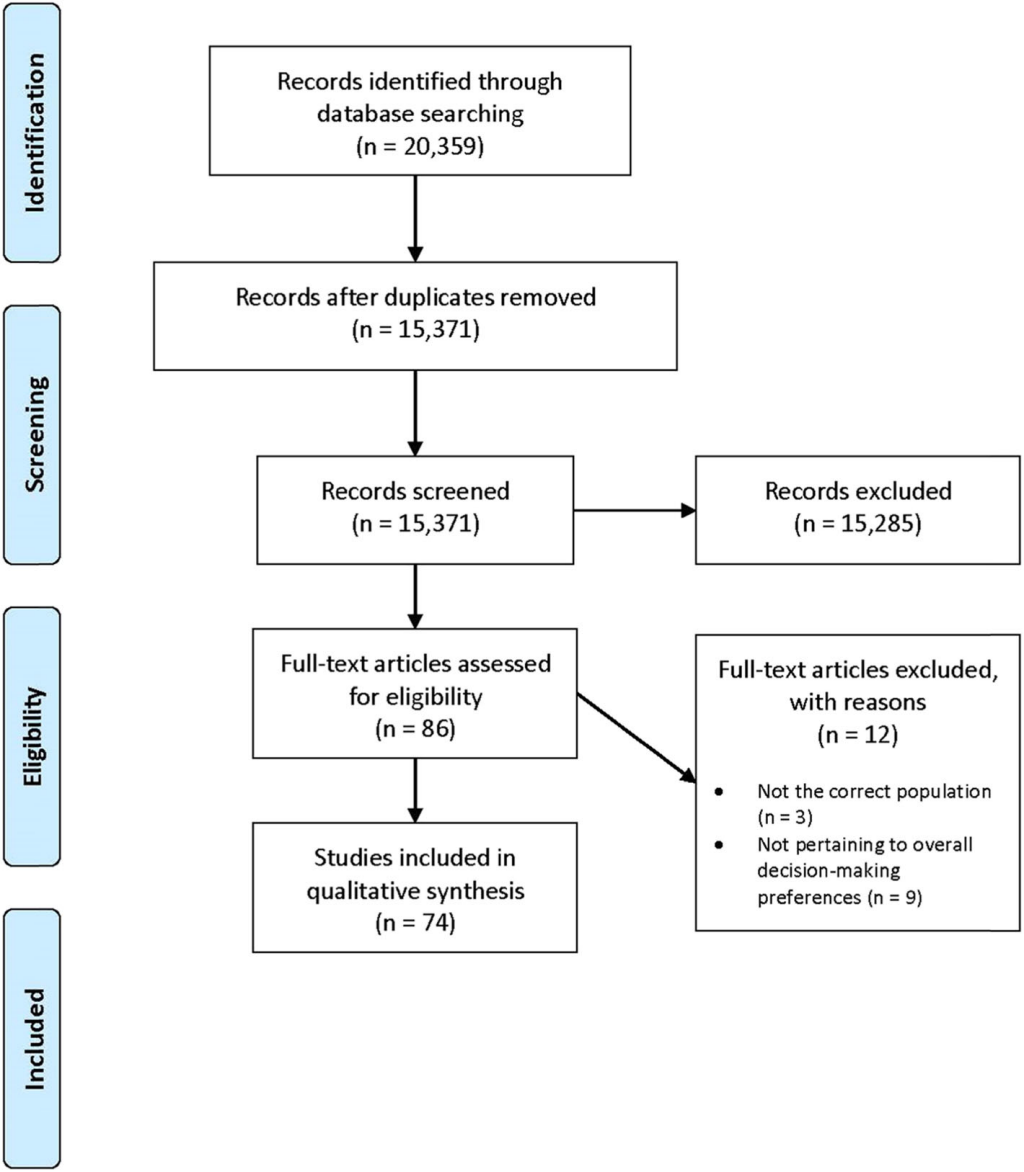
Flow diagram of study selection

After ascertaining our research questions, we worked in conjunction with an experienced medical librarian to identify relevant studies. We followed the Preferred Reporting Items for Systematic Reviews and Meta-analyses (PRISMA) guidelines for reporting the identified, screened, eligible, and included studies (Fig. 1). After drafting, refining, and finalizing our search strategies, we searched three bibliographic databases from inception through November 2017: Medline, EMBASE, and Cochrane databases. The final search strategies for all three databases are outlined in Additional file 1. The final search results were imported into Endnote (version X9.1, 2019) and yielded 20,359 articles.

### Study design

#### Eligibility criteria

Inclusion and exclusion criteria were defined a priori. Review was limited to English language (no translators available), peer-reviewed, published literature. Only empirical studies were included. Review was limited to decision making preferences of surgeons and/or adult patients. Decision making preferences were loosely defined and included “preferred role,” “perceived role,” “expectations,” “desires,” and “satisfaction with actual decision-making role.” Articles in the following categories were excluded: reviews, letters to the editor, editorials, suggested models of care, patient education handouts, decision making tools, animal studies, and articles related to pediatric surgery. In addition, we excluded articles without accessible full text.

#### Literature review

After duplicates were removed by the primary author (LAS), we were left with 15,371 articles to screen. Two of the authors (LAS and EMC) independently reviewed all titles and abstracts and jointly decided to exclude 15,285 articles based on the eligibility criteria. The remaining 86 articles were selected for full text review. Following full text review, 12 additional articles were excluded because they either did not pertain to an adult surgical population or to decision-making preferences in the surgery setting. Disagreements were resolved by discussion between the two authors.

### Charting the data

For each of the 74 included articles, two of the authors (LAS and CJK) independently abstracted the following outcome measures: study methods (quantitative/qualitative/mixed methods), surgical specialty, cancer diagnosis (yes/no/unclear), study location (US/non-US), study setting (inpatient/outpatient), type of subject (patient/surgeon), number of subjects, gender, acuity of the intervention (elective/urgent/emergent/unclear), surgical decision to be made (surgery v non-operative management/choice among different surgical procedures/decision on timing of surgery/other), surgeon/patient decision making preferences (shared decision making/ surgeon guided decision making/ independent decision making), and surgeon/patient factors associated with favoring SDM.

Acuity of the intervention was defined as follows: emergent (immediate need for surgery to preserve life); urgent (surgery is required within the next days or weeks); and elective (surgery is not required). Notably, cancer resections were considered urgent, however subsequent reconstruction was considered elective (i.e. breast cancer resection with subsequent reconstruction). The Control Preferences Scale, which is a five-point measure used to gauge preferred involvement in medical decision making [16], was adapted to define surgeon/patient preferences as follows: *Shared decision making (SDM)*: the patient and surgeon prefer to make the decision regarding surgery together; *Surgeon guided decision making (SG)*: the preference is for the surgeon to guide decision making (either entirely or in part) while the patient takes a more “passive role;” *Independent decision making (IDM)*: the preference is for the patient to take a more “active role” in decision making (either partly or entirely) independent from the surgeon.

The data abstraction form was a modified version of the one we used for a literature review we conducted on decision making preferences in the pediatric surgical setting [13]. Discordant opinions were discussed at weekly meetings. The third author (EMC) was available to mediate if consensus could not be reached. Data was analyzed in Microsoft Excel (2019).

## Results

### Summarizing, collating, and reporting the results

#### Summarizing the results

20,359 articles were identified (Medline *n* = 10, 665; Embase *n* = 9036; Cochrane *n* = 658). 4988 duplicates were removed, and 15,371 articles underwent title/abstract review. Seventy-four articles were included in the final analysis because they specifically addressed existing empirical evidence about patient and/or surgeon decision making preferences toward SDM in adult surgery setting. Table 1 provides a summary of all included articles.

**Table 1:** Characteristics of 74 included articles

Article and Date of Publication	Study Population	Acuity of the Intervention	Major Findings Related to Decision Making (DM) Preferences	DM Theme Related to Major Findings^a^		Factors Associated with Favoring SDM
				^PT	^SURG	
Almyroudi et al. (2011) [17]	329 breast cancer patients	Urgent	71.1% preferred a passive role; 24% a collaborative role;4.6% an active role	SG	–	Younger age, higher education
Ananian et al. (2004) [18]	181 breast cancer patients	Elective	57% of women choosing breast reconstruction “decided with surgeon” 70% of these patients were satisfied with the information received.	SDM	–	Type of procedure
Andersen et al. (2009) [19]	636 breast cancer survivors	Urgent	On average, 72% reported being “very involved, I made all the decisions myself.” 80% were content with DM role.	IDM	–	Younger age, level of education, income
Asghari et al. (2008) [20]	299 hospitalized patients (85% on surgical wards)	Unclear	“strongly desire to receive information and participate in decision-making”	SDM	–	Female, level of education
Ashraf et al. (2013) [21]	465 patients undergoing either immediate or delayed breast reconstruction	Elective	66% were in the “informed-consumerist” group when it came to actual DM. 86.3% of these patients were satisfied with the information received.	IDM	–	
Avis (1994) [22]	20 hernia repair patients	Elective	“expectations of participation can be summarized as ‘being told’ and ‘going in to get it fixed’”	SG	–	
Ballinger et al. (2008) [23]	131 breast cancer patients	Urgent	61% “felt their healthcare professionals had surgical preferences for them, believed that clinical issues determined these preferences, but still knew the choice was theirs”	SDM	–	
Beaver et al. (2005) [24]	41 colorectal cancer patients	Urgent	“wanted to be well informed and involved in the consultation process but did not necessarily want to use the information they received to make decisions”	SG	–	
Beaver et al. (2007) [25]	35 health professionals caring for colorectal cancer patients (4 were surgeons)	Urgent	“shared decision making was favored by health professionals”	–	SDM	Younger patient age
BeLue et al. (2004) [26]	50 cardiologists making a decision about surgery; 92 patients with coronary artery disease	Urgent	Physicians: 74% “prefer patients who actively participate in the decision;” Patients: 50% “prefer the physician to make the decision;” 40% SDM;” 10% “prefer to make the decision on their own”	SG	SDM	
Blumenthal-Barby et al. (2015) [27]	30 left ventricular assist device patients and candidates	Urgent	“deferred heavily to clinicians”	SG	–	
Burton et al. (2017) [28]	101 older breast cancer patients	Urgent	39% preferred “patient-centred;” 38% “doctor-centred;” 24% SDM	SG/ IDM	–	
Butow et al. (2007) [29]	135 patient advocates; 142 breast cancer surgeons	Urgent	66% of surgeons and 62% of patient advocates preferred SDM	SDM	SDM	
Campesino et al. (2012) [30]	39 breast cancer survivors	Urgent	Spanish-speaking Latinas preferred “physician treatment recommendations;” English-speaking Latinas and African-Americans preferred SDM	SDM/ SG	–	English-speaking
Cohen (2003) [31]	19 patients with localized prostate cancer	Urgent	Most viewed the surgeon-guided approach as “appropriate and welcome.”	SG	–	
Corriere et al. (2015) [32]	81 patients undergoing elective vascular procedures	Elective	93% preferred “choosing together with the provider;” 62% preferred “having the provider choose for them”	SDM	–	Multiple treatment options, type of procedure
Cuypers et al. (2016) [33]	562 prostate cancer survivors	Urgent	59% preferred a collaborative role; 22% an active role; 19% a passive role	SDM	–	Higher education; younger age; higher SES
Doring et al. (2014) [34]	105 hand surgeons; 84 patients with trigger finger	Elective	Patients “preferred to decide for themselves”; surgeons preferred SDM	IDM	SDM	
Durif-Bruckert et al. (2015) [35]	146 breast cancer patients	Urgent	wanted to participate in decisions, but “perceived SDM as an obligation” because it did not seem to fit with their idea of a proper doctor-patient relationship	SG	–	Trust in surgeon; support from family; written information from surgeon
Gainer et al. (2017) [36]	15 frail and older patients; 20 care team members (includes surgeons)	Unclear	both patients and care team members “supported a formal approach” to SDM	SDM	SDM	
Ghane et al. (2014) [37]	380 general surgery patients	Elective	“preferred relatively high levels of decisional control on average (M = 8.95 out of 10, SD = 2.15).”	IDM	–	Male; good health; high health literacy
Golden et al. (2017) [38]	20 clinicians (7 were surgeons)	Urgent	Most felt that they practiced SDM, even though they did not tend to distinctly prompt patient DM preferences	–	SDM	
Gong et al. (2011) [39]	78 patients with carpal tunnel syndrome	Elective	76% preferred SDM	SDM	–	History of surgical procedure; importance of family member opinions; having private insurance
Hack et al. (2006) [40]	205 breast cancer patients	Urgent	42% preferred a collaborative role; 35.6% an active role; 22.4% a passive role	SDM	–	Age < 70, non-widowed, longer duration post-op
Hageman et al. (2014) [41]	103 hand surgeons; 79 patients with carpal tunnel syndrome	Elective	Surgeons: 74% preferred “patient and provider make a shared decision;” Patients: 59% preferred that “the patient decides”	IDM	SDM	
Hawley et al. (2008) [42]	925 breast cancer patients	Urgent	Actual DM role: 37% SDM; 36% “patient-based;” 27% “surgeon-based.” Preferred DM role: 93% content with level of DM involvement	SDM/ IDM	–	
Heggland & Hausken (2013) [43, 44]	11 health professionals from 6 surgical wards; 7 patients who underwent surgical treatment	Elective	Health professionals: majority preferred a “shared” or “informed” model; Patients: about half preferred a “shared” or “informed” model and the other half preferred a “paternalistic” model	SDM/ SG	SDM	Female
Heggland & Hausken (2014) [45]	7 surgical patients; 4 surgeons	Elective/ Urgent	Surgeons: the majority preferred an “informed model … patient is given information and left to make the decision;” Patients: 3 preferred a “paternalistic model” and 2 preferred shared.	SG	IDM	
Heggland et al. (2014) [44, 45]	119 physicians working in 6 surgical wards	Unclear	physicians on average rated decision-making control a 4.6, which means that “physicians were not reluctant to involve patients in decision-making processes”	–	SDM	
Henderson & Shum (2003) [46]	49 surgical and medical patients	Elective/ Urgent	Where 1 = active role, 3 = shared, and 5 = passive – the mean DM value for the severe scenario was 3.55; moderate scenario was 3.37; mild scenario was 3.00	SDM	–	Younger age, non-critical condition
Henderson et al. (2006) [47]	186 inpatients in two surgical units	Unclear	“females indicated that they would like to have more input in the decision-making process than the males” (3.57 v. 3.81 on the Controlled Preferences Scale)	SDM	–	Female; higher education
Hopmans et al. (2015) [48]	87 lung cancer patients	Urgent	“guidance by the clinician” was identified as most important; “active role of patient in treatment decision making” regarded as less important	SG	–	
Hou et al. (2014) [49]	113 colorectal cancer patients	Urgent	41.6% preferred a passive role; 24.8% SDM; 7.1% an active role	SG	–	Female; no stoma
Iaccarino et al. (2017) [50]	428 clinician members of the American Thoracic Society	Urgent	Perceived Role: 50.4% “share decisions equally with the patient”; 34.5% “allow the patient to decide;” 15.1% “decide for themselves after considering the patient’s opinion”	–	SDM	More years in practice; more comfort in pulmonary nodule management
Ihrig et al. (2011) [51]	31 prostate cancer patients	Urgent	“most patients wanted to decide on their treatment options together with their physician”	SDM	–	
Janz et al. (2004) [52]	101 breast cancer patients	Urgent	47% preferred SDM; 38% preferred to make the decision “with physician input”	SDM	–	College degree; higher self-efficacy
Johnson et al. (1996) [53]	76 newly diagnosed breast cancer patients	Urgent	“74% wanted their surgeons to make a recommendation and when given, 94% followed the recommended treatment plan”	SG	–	
Keating et al. (2002) [54]	1081 breast cancer patients	Urgent	64% preferred a collaborative role	SDM	–	
Keating et al. (2010) [55]	5383 lung or colorectal cancer patients	Urgent	38.9% = “patient controlled,” 43.6% = SDM; 17.5% = “physician controlled”	SDM	–	Married, better pre-diagnosis health status, Caucasian, strong evidence for procedure
Lally (2009) [56]	18 breast cancer patients	Urgent	“women’s lack of sharing their preferences with their surgeons and the surgeons’ lack of making treatment recommendations resulted in what was more likely *informed* than *shared* decision making”	IDM	–	
Lam et al. (2003) [57]	154 breast cancer patients	Urgent	59% preferred SDM; 33% preferred “the choice to be their own;” 8% preferred “to delegate the decision”	SDM	–	Younger age
Lantz et al. (2005) [58]	1633 breast cancer patients	Urgent	Actual Role: 36.9% SDM; 37.9% made decision with “surgeon input.” 69% were satisfied with DM level.	SDM	–	
Larsson et al. (1989) [59]	666 patients scheduled for invasive surgery	Elective	Actual DM: 41% “joint patient-doctor decision;” 29% “doctor advocated;” 8% “patient asked.” Preferred DM: 73% content with level of DM involvement	SDM	–	Female
Lee et al. (2012) [60]	82 patients with early gastric cancer	Urgent	The surgical group showed a more passive role in both their preferred and actual DM role	SG	–	
Markovic et al. (2006) [61]	30 newly diagnosed gynecologic cancer patients	Urgent	“surgeon’s recommendation and fear of dying from cancer” played the most important role in DM	SG	–	
Martinez et al. (2016) [62]	1690 newly diagnosed breast cancer patients	Urgent	In surgery, 51% preferred a “directive” communication style; 49% a “non-directive” communication style	SDM/ SG		
McGuire et al. (2005) [63]	18 surgeons	Unclear	“Many physicians saw their role as an expert who educates the patient but retains control over the decision-making process; others took a more collaborative approach, encouraging patients to assume decisional priority”	–	SDM/ SG	Multiple treatment options, increased risk, impact of procedure on patient lifestyle, moral content
Mendick et al. (2010) [64]	20 breast cancer patients; 8 surgeons	Urgent	Surgeons: “made most decisions for patients;” Patients: “generally lacked trust in their own decisions and usually sought surgeons’ guidance”	SG	SG	Patients: strong evidence for procedure; Surgeons: multiple treatment options, impact of procedure on patient lifestyle
Meredith (1993) [65]	30 surgical patients; 14 surgeons	Unclear	Patients: “majority agreed that the surgeon should supply them with the ‘pros’ and ‘cons’ of all measures to address the problem, and it was for them ultimately to decide what was right for them;” Surgeons: “not enthusiastic at the prospect of devoting more time to discussing surgical alternatives, risks and complications, and outlook indicators for their patients benefit”	SDM	SG	
Morgan et al. (2015) [66]	729 older breast cancer patients	Urgent	In surgery, 41.6% preferred SDM; 34.7% a “doctor-centered” approach; “23.7% a “patient-centered” approach	SDM	–	Older age
Morishige et al. (2017) [67]	1035 patients with irritable bowel disease	Elective	56% “thought having a physician involve them in the decisions concerning their treatment was very important”	SDM	–	Comorbidities, surgical history; use of biologics, treated at an academic hospital, being married
Moumjid et al. (2003) [68]	22 breast cancer patients	Urgent	**“**most were satisfied with the information given and the possibility of participating to the treatment decision-making process”	SDM	–	
Nam et al. (2014) [69]	85 patients with carpal tunnel syndrome	Elective	“I prefer that my doctor and I share responsibility” = 29%; ““I prefer that my doctor makes the final decision about which treatment will be used but seriously considers my opinion = 35%	SDM	–	
Omar et al. (2016) [70]	100 consecutive patients being seen in a multi-disciplinary stone clinic	Elective	85% “would rely on the physician’s recommendation”	SG	–	
Op den Dries et al. (2014) [71]	219 liver transplant candidates and recipients	Urgent	“79.8% wished to be involved in making the decision to accept or not accept a liver for transplantation”	SDM	–	
Orsino et al. (2003) [72]	197 end stage renal disease patients	Elective	41.5% preferred “equal responsibility;” 34.5% an “autonomous” role; 23.9% a decision driven by the health care team	SDM	–	Younger age
Pieterse et al. (2008) [73]	70 rectal cancer patients; 25 surgical oncologists	Urgent	The majority of patients and clinicians preferred SDM.	SDM	SDM	Patients: Female, higher education
Ramfelt et al. (2005) [74]	55 rectal or colon cancer patients	Urgent	71% of rectal cancer patients & 75% of colon cancer patients preferred a collaborative role	SDM	–	Younger age
Ratsep et al. (2014) [75]	150 patients with lumbar disc herniation	Elective	47% preferred SDM	SDM	–	Desire for more disease specific information
Salkeld et al. (2004) [76]	175 rectal or colon cancer patients	Urgent	54% preferred a surgeon-guided approach; 29% SDM; 15% a more independent DM role	SG	–	Female, younger age, history of radiation
Santema et al. (2017) [77]	67 patients with either abdominal aortic aneurysm or peripheral arterial occlusive disease	Elective	58% preferred SDM	SDM	–	Trust in doctor, doctor has a clear communication style, doctor listens, enough time for consultation
Seror et al. (2013) [78]	415 young breast cancer patients	Urgent	Preferred a more passive approach (20.7% preferred “fully passive” and 36.4% preferred fairly passive)	SG	–	
Sidana et al. (2012) [79]	488 young prostate cancer patients	Urgent	52.3% preferred SDM; 45.8% an “informed decision made by myself based on information”; 2% a passive role	SDM	–	Higher education, type of procedure
Snijders et al. (2014) [80]	103 GI surgeons	Urgent	“most patients were offered only one treatment option and little SDM was seen”	–	SG	
Stiggelbout & Kiebert (1997) [81]	52 cancer patients; 48 surgical patients	Unclear	“the physician should make the decisions, but strongly consider my opinion” was selected most frequently	SG	–	Younger age, female
Sung et al. (2010) [82]	93 patients with pelvic floor disorder	Elective	47% preferred a collaborative role; 44% an active role; 9% a passive role	SDM	–	
Tyler Ellis et al. (2016) [83]	154 newly diagnosed rectal cancer patients	Urgent	43% of total mesorectal excision patients and 44% of local excision patients preferred SDM	SDM	–	Higher education, younger age
Uldry et al. (2013) [84]	253 patients undergoing elective GI surgery	Elective	64% preferred an active role	IDM		Younger age, male, level of education
Vogel et al. (2008) [85]	137 breast cancer patients	Urgent	40.2% preferred a passive role; 30.6% an active role; 29.2% SDM	SG	–	Higher anxiety scores; multiple treatment options
Wang et al. (2018) [86]	154 breast cancer patients	Urgent	55.2% preferred a collaborative role; 27.5% a passive role; 17.5% an active role	SDM	–	
Weiner & Essis (2006) [87]	100 spine clinic patients	Elective	“the majority of patients felt that the physician, rather than the patient, should make the basic treatment decision”	SG	–	
Wilson et al. (2017) [88]	157 patients undergoing major thoracic/abdominal operations	Urgent	65.4% preferred a “patient-driven” role; 28.8% SDM; 5.8% a “surgeon-driven” role	IDM	–	
Woltz et al. (2017) [89]	50 patients with displaced midshaft clavicular fracture	Elective	36% preferred SDM; 34% “autonomous” role; 30% a passive role	SDM	–	
Ziebland et al. (2006) [90]	43 ovarian cancer patients	Urgent	“preferred their medical team to decide on their behalf” or “‘going along with’ their doctor’s recommendation”	SG	–	

^a^Decision Making Preference: *DM* decision making, *SG* surgeon-guided, *SDM* shared decision making, *IDM* independent decision making

^^^*Dx* Diagnosis, *Pt* Patient, *Surg* Surgeon

#### Collating and reporting the results

Table 2 provides frequencies for the characteristics of all included articles. Over half of the articles were quantitative (*n* = 49; 66%) and performed outside of the US (*n* = 48; 65%). Sixty-seven (91%) included outpatient surgeries. Fourteen surgical subspecialties were represented with the most articles originating from Surgical Oncology (*n* = 29; 39%), General Surgery (*n* = 13; 18%), Orthopedic Surgery (*n* = 10; 14%), and Urology (*n* = 9; 12%). Fifty (68%) articles discussed decision making for patients with cancer, and 23 (46%) of these focused on breast cancer. Most articles assessed a choice between operative and non-operative management (*n* = 37; 50%) or an option among different surgical procedures (*n* = 29; 39%).

**Table 2:** Frequencies for characteristics of all included articles (*n* = 74)

Variable	Studies, n(%)
Surgical specialty^a^	
Oncology	29 (39)
General Surgery	13 (18)
Orthopedics	10 (14)
Urology	9 (12)
Gynecology	7 (9)
Colorectal	6 (8)
Thoracic	6 (8)
Cardiac	5 (7)
Plastic Surgery	4 (5)
Transplantation	3 (4)
Vascular	3 (4)
Neurosurgery	2 (3)
ENT/Otolaryngology	1 (1)
Ophthalmology	1 (1)
Cancer diagnosis	
Yes	50 (68)
No	19 (26)
Unclear	5 (7)
Study methods	
Qualitative	18 (24)
Quantitative	49 (66)
Mixed methods	7 (9)
Study location	
US	26 (35)
Non-US	48 (65)
Study setting	
Inpatient	7 (9)
Outpatient	64 (86)
Both	3 (4)
Type of subjects	
Patients only	58 (78)
Surgeons only	6 (8)
Both patients and surgeons	10 (14)
Number of subjects	
1–5	1 (1)
6–20	7 (9)
21–50	12 (16)
51–100	11 (15)
101–500	33 (45)
> 501	10 (14)
Population gender	
Male only	4 (5)
Female only	25 (34)
Both	45 (61)
Clinical dilemma	
Surgery versus non-operative management	37 (50)
Choice among surgical procedures	29 (39)
Timing of surgery	4 (5)
Other	4 (5)
Acuity of intervention^a^	
Elective	22 (30)
Urgent	47 (64)
Emergent	0 (0)
Unclear	7 (9)
Surgeon preference	
Favors surgeon-guided decision making	4 (25)
Favors shared decision making	12 (75)
Favors independent decision making	0 (0)
Patient preference	
Favors surgeon-guided decision making	26 (35)
Favors shared decision making	40 (54)
Favors independent decision making	8 (11)

^a^Overlap exist among surgical specialties, acuity of intervention, and patient preference resulting in % > 100

Sixty-eight (92%) of the articles included patients. Of these, 40 (54%) demonstrated that patients preferred SDM, 26 (35%) showed that patients favored a surgeon-guided approach, and 8 (11%) revealed a patient preference for independent decision making. The most common factors for patients favoring SDM included female gender, higher education, and younger age.

Only 16 (22%) of the articles assessed surgeons’ preferences. Of these, 12 (75%) found that surgeons preferred SDM, while 4 (25%) demonstrated that surgeons favored a more surgeon-guided decision-making approach. The factors most commonly listed for surgeons favoring SDM included limited evidence for a given treatment plan, multiple treatment options, and impact on patient lifestyle.

None of the articles evaluated patient decision-making preferences in an emergent setting. Out of the 22 articles that assessed patient decision-making preferences in the elective surgery setting, 13 (59%) preferred SDM. Three out of four (75%) of the articles assessing surgeon decision making preferences in the elective surgery setting reported that surgeons preferred SDM. In six out of nine (67%) of the articles, surgeons also preferred SDM in the urgent surgery setting. In 47 articles, patients were fairly split on their decision-making preference when it came to urgent surgeries with 47% desiring SDM and 43% favoring a more surgeon-guided approach.

Only 10 articles (14%) looked at both patient and surgeon decision making preferences. In a little over half of these articles (*n* = 6; 60%), there was discordance between patient and surgeon decision making preferences. Out of these articles, three focused on elective surgeries in Orthopedics, one on urgent surgeries in Cardiac Surgery, one on both elective and urgent surgeries in General Surgery, and one was unclear on the acuity of the invention but occurred in General Surgery.

## Discussion

Shared decision making has been highlighted as a desirable approach to clinical counseling [1]. However, it is unclear if this applies to surgical decision making, particularly when considering surgical counseling in settings of emergent, complex, highly-morbid operations [13]. In our scoping review of the adult surgical literature, we found relatively few studies that address patient and surgeon preferences toward SDM in surgery. We found that a large proportion of existing articles on preferences toward SDM address elective, outpatient procedures. While patients did seem to prefer SDM in these controlled settings, it is possible that patients and surgeons may prefer more surgeon guidance when discussing emergent, complex operations that have a high risk of morbidity or mortality. Further studies that specifically target decision making regarding complex, emergent procedures should be performed to help surgeons develop a more nuanced understanding of patient preferences and expectations in these highly-charged clinical encounters. A more refined approach to such potentially challenging surgical counseling may enhance trust, which has been shown to predict satisfaction with care and overall adherence to treatment plans [91].

Our finding that no studies evaluated SDM in emergent surgical settings likely exemplifies the presumed difficulty with engaging patients and surrogates in SDM in emergent, life-threatening settings where there is limited time to evaluate options, absorb information, or deliberate over alternatives in a way that affords the opportunity to make sensible decisions [92, 93]. However, even in the most dire circumstances, there is usually time to have some discussion with patients and surrogates that adheres to the goals of SDM [92]. The conversation may certainly be different than it would when one is engaging a patient in SDM regarding an elective procedure, but most emergencies do not preclude the opportunity for some discussion. The options presented may include only surgery or death, but deciding between the two may require patient/surrogate consideration of the possible outcomes associated with surviving surgery-including post-operative dialysis, paralysis, or dependence on skilled nursing care [94]. These preference-based decisions suggest that SDM may be meaningful in such settings [92, 94]. Yet, there are no published studies addressing whether it is the preference of patients or surgeons to engage in SDM in the emergent surgical setting.

There may be ethical challenges with conducting such studies that account for this lack of data. Some may question the appropriateness of asking patients or surrogates to pause and reflect upon decision making preferences during an acute health crisis [92]. Development of studies that aim to retrospectively evaluate patients’ and surrogates’ attitudes toward decision making preferences after the acuity of a given situation has lessened may be an ethically feasible means by which to investigate this issue. Future investigation should also include consideration of the impact of advanced care planning on decision making preferences in emergent settings. Assuring that a patients’ goals, values, and preferences are clearly articulated and documented prior to finding him or herself in the often-unexpected position of needing an urgent surgical intervention may improve the decision-making process [95].

Additionally, the majority of articles we identified in this review assessed patient preferences toward SDM, but very few evaluated preferences of surgeons toward SDM. Inclusion of surgeon preferences in future studies is critical to assure that counseling strategies that incorporate surgeon insight and preferences are developed. Failure to include surgeon perspectives in this discussion limits the eventual integration of recommendations into surgical practice. Future work should also strive to gain an understanding of whether surgeon preferences regarding their and their patients’ roles in decision making vary over the course of a surgeon’s career. One may speculate that surgeons prefer to be more directive in patient counseling as their careers and level of experience progress, but there has been limited investigation into whether such a trend exists [50]. A more robust understanding of surgeon preferences would aid in the development of clinical counseling training programs for junior surgeons and trainees as well as continuing medical education programs for senior surgeons. An understanding of surgeon preferences toward SDM is needed to assure surgeon engagement and buy-in into such clinical training programs.

Our review suggests that Surgical Oncology has been the most active surgical subspecialty in the investigation of surgeons’ and patients’ preferences toward SDM. Much of this work has involved decision-making regarding breast cancer, and these articles have generally shown that breast cancer patients prefer SDM [18, 23, 40, 42, 52, 54, 57, 58, 66, 68, 86]. However, the meaning of patients’ expressed preferences toward SDM in surveys has been called into question by some authors [35]. In a study of breast cancer patients, Durif-Bruckert et al. found that the majority of patients stated that they preferred SDM when asked via survey [35]. However, when asked about the process of decision making in a qualitative interview, many of the same patients expressed that they did not understand the medical details, felt overwhelmed by the discussion with the surgeon, and essentially desired more guidance from their surgeon [35]. The authors speculate that patients may confuse “participation” with true SDM, thus calling into question much of the survey-based data on patient preferences toward SDM [35]. Such a finding is critical, as the majority of existing studies on this topic utilize survey instruments to assess patient preferences.

The idea that patients may prefer “participation” as opposed to true SDM was highlighted in several other articles identified in our search [24, 64, 81, 87]. Beaver et al. found that while colorectal cancer patients wanted to be well informed and involved in their care, they did not want to make final treatment decisions [24]. Weiner and Essis also found that patients considering spine surgery desired detailed information regarding operative interventions, but they preferred that the surgeon make the final decision regarding surgery [87]. Stiggelbout and Kiebert echoed similar findings in their evaluation of the decision-making preferences of cancer patients [81]. Overall, the authors found that patients preferred their physicians make the treatment decisions with consideration of the patients’ opinion. Consistent with the previously mentioned studies, even those patients who desired more information during surgical consultation, preferred their surgeon make the decisions regarding treatment [81]. Interestingly, in a study of breast cancer patients, Mendick et al. found that patients’ preferences for guidance during the decision-making process stemmed from a lack of trust in their own decision-making abilities [64]. Despite this, patients expressed that their engagement in discussion with the surgeons, as well as the opportunity to refuse recommendations, gave them a sense of ownership of the decisions made by the surgeons [64]. These studies reinforce the idea that a patients’ desire for participation and engagement in the decision-making process does not necessarily imply a desire for shared decision making. Future work in the field should thus strive to assure that the true meaning of SDM is captured in the assessment tools. Studies that utilize qualitative methods or mixed methods approaches may offer a better means to clarify the specific facets of decision making that are most important to patients.

The abundance of studies of SDM in breast cancer patients may also skew the already limited literature on surgical SDM in that it results in more female patients being evaluated than male patients. In our analysis, we found that being female was one of the key factors associated with preferring SDM. The relatively large number of studies of decision making in breast cancer patients within this body of literature may thus create a false impression of the proportion of patients who generally prefer SDM. Assuring that decision-making preferences are assessed in both male and female patients, as well as in clinical settings predominantly experienced by men, will help address this potentially confounding issue.

In contrast to our prior review of decision-making preferences in Pediatric Surgery where Otolaryngology had performed the majority of studies (specifically related to cochlear implants) [13], Otolaryngology as a field had very few studies in adult decision-making preferences. This suggests that certain procedures such as cochlear implantation or breast cancer resection and reconstruction may seem particularly suited for SDM. However, assuring that patient and surgeon preferences are considered across a wide spectrum of pathology will allow the most refined insight into true decision-making preferences.

The majority of articles identified here highlight decision making regarding the choice between operative or non-operative management or a choice among different surgical procedures. Inclusion of issues such as timing of surgery or the need for inpatient as opposed to outpatient post-operative management, would offer a more robust understanding of overall preferences toward SDM. Our study also highlights that the majority of identified studies were not performed in the US. It is likely that international perspectives toward surgeon guidance and healthcare delivery may have impacted our results. A more detailed global perspective on patient preferences toward SDM could be achieved by performing comparative investigation of preferences across countries.

Our work has several limitations. We did not incorporate unpublished data, such as abstracts presented at society meetings, in our study. This may have limited the number of articles we identified. Despite this potential limitation, our approach involved reviewing over 15,000 articles, which may have prompted reviewer fatigue. To limit the impact of reviewer fatigue and to minimize potential reviewer bias, two independent reviewers assessed each article and a third reviewer was available to resolve disagreements. Additionally, our search strategy was specialty based (i.e. surgery) as opposed to pathology based (i.e. prostate cancer), and it is possible that designing our search in this manner resulted in failure to include studies that offer predominantly medical, but occasionally surgical, treatment options.

## Conclusions

Limited data regarding patient and surgeon preferences toward shared decision making exists in the surgical literature. Generally, patients and surgeons expressed preference toward SDM. For patients, female gender, higher education, and younger age were associated with a preference for SDM. Surgeons favored SDM in settings that included limited evidence for a given treatment plan, multiple treatment options, and impact on patient lifestyle. None of the articles evaluated decision-making preferences in an emergent setting, so assessment of the impact of acuity of intervention on decision making preferences is limited. Most available articles focus on non-emergent, outpatient decision making related to oncology. Further research is needed to better understand the range of preferences surgeons and patients have regarding SDM across diverse clinical settings. Extension of this research to non-oncologic, complex, and emergent clinical settings is particularly needed.

## Supplementary information

**Additional file 1.** Search strategies for Medline, Embase, and Cochrane databases.

## Data Availability

The datasets used and/or analyzed during the current study are available from the corresponding author on reasonable request.

## Abbreviations

SDM: Shared decision making
SG: Surgeon guided decision making
IDM: Independent decision making
PT: Patient
SURG: Surgeon

## References

[1] Elwyn G, Frosch D, Thomson R, Joseph-Williams N, Lloyd A, Kinnersley P (2012). Shared decision making: a model for clinical practice. J Gen Intern Med.

[2] Braddock CH (2010). The emerging importance and relevance of shared decision making to clinical practice. Med Decis Mak.

[3] Boss EF, Mehta N, Nagarajan N, Links A, Benke JR, Berger Z (2016). Shared decision making and choice for elective surgical care: a systematic review. Otolaryngol Head Neck Surg.

[4] Frosch DL, Moulton BW, Wexler RM, Holmes-Rovner M, Volk RJ, Levin CA (2011). Shared decision making in the United States: policy and implementation activity on multiple fronts. Z Evid Fortbild Qual Gesundhwes.

[5] Ploug T, Holm S (2015). Doctors, patients, and nudging in the clinical context--four views on nudging and informed consent. Am J Bioeth.

[6] Lantos JD (2015). Do patients want to participate in decisions about their own medical care?. Am J Bioeth.

[7] Rosenbaum L (2015). The paternalism preference--choosing unshared decision making. N Engl J Med.

[8] Robinson A, Thomson R (2001). Variability in patient preferences for participating in medical decision making: implication for the use of decision support tools. Qual Health Care.

[9] Elkin EB, Kim SH, Casper ES, Kissane DW, Schrag D (2007). Desire for information and involvement in treatment decisions: elderly cancer patients' preferences and their physicians' perceptions. J Clin Oncol.

[10] Strull WM, Lo B, Charles G (1984). Do patients want to participate in medical decision making?. JAMA..

[11] Degner LF, Sloan JA (1992). Decision making during serious illness: what role do patients really want to play?. J Clin Epidemiol.

[12] Pollard S, Bansback N, Bryan S (2015). Physician attitudes toward shared decision making: a systematic review. Patient Educ Couns.

[13] Carlisle EM, Shinkunas LA, Kaldjian LC (2018). Do surgeons and patients/parents value shared decision- making in pediatric surgery? A systematic review. J Surg Res.

[14] Arksey H, O’Malley L (2005). Scoping studies: towards a methodological framework. Int J Soc Res Methodol.

[15] Tricco AC, Lillie E, Zarin W (2018). Prisma extension for scoping reviews (PRISMA-ScR): checklist and explanation. Ann Intern Med.

[16] Degner LF, Sloan JA, Venkatesh P (1997). The control preferences scale. Can J Nurs Res.

[17] Almyroudi A, Degner LF, Paika V (2011). Decision-making preferences and information needs among Greek breast cancer patients. Psychooncology..

[18] Ananian P, Houvenaeghel G, Protiere C (2004). Determinants of patients' choice of reconstruction with mastectomy for primary breast cancer. Ann Surg Oncol.

[19] Andersen MR, Bowen DJ, Morea J (2009). Involvement in decision-making and breast cancer survivor quality of life. Health Psychol.

[20] Asghari F, Mirzazadeh A, Fotouhi A (2008). Patients' preferences for receiving clinical information and participating in decision-making in Iran. J Med Ethics.

[21] Ashraf AA, Colakoglu S, Nguyen JT (2013). Patient involvement in the decision-making process improves satisfaction and quality of life in postmastectomy breast reconstruction. J Surg Res.

[22] Avis M (1994). Choice cuts: an exploratory study of patients' views about participation in decision-making in a day surgery unit. Int J Nurs Stud.

[23] Ballinger RS, Mayer KF, Lawrence G (2008). Patients' decision-making in a Uk specialist Centre with high mastectomy rates. Breast..

[24] Beaver K, Jones D, Susnerwala S (2005). Exploring the decision-making preferences of people with colorectal cancer. Health Expect.

[25] Beaver K, Craven O, Witham G (2007). Patient participation in decision making: views of health professionals caring for people with colorectal cancer. J Clin Nurs.

[26] BeLue R, Butler J, Kuder J (2004). Implications of patient and physician decision making: an illustration in treatment options for coronary artery disease. J Ambul Care Manage.

[27] Blumenthal-Barby JS, Kostick KM, Delgado ED (2015). Assessment of patients' and caregivers' informational and decisional needs for left ventricular assist device placement: implications for informed consent and shared decision-making. J Heart Lung Transplant.

[28] Burton M, Kilner K, Wyld L (2017). Information needs and decision-making preferences of older women offered a choice between surgery and primary endocrine therapy for early breast cancer. Psychooncology..

[29] Butow P, Harrison JD, Choy ET (2007). Health professional and consumer views on involving breast cancer patients in the multidisciplinary discussion of their disease and treatment plan. Cancer..

[30] Campesino M, Koithan M, Ruiz E (2012). Surgical treatment differences among Latina and African American breast cancer survivors. Oncol Nurs Forum.

[31] Cohen H, Britten N (2003). Who decides about prostate cancer treatment? A qualitative study. Fam Pract.

[32] Corriere MA, Avise JA, Peterson LA (2015). Exploring patient involvement in decision making for vascular procedures. J Vasc Surg.

[33] Cuypers M, Lamers RED, de Vries M (2016). Prostate cancer survivors with a passive role preference in treatment decision-making are less satisfied with information received: Results from the profiles registry. Urol Oncol.

[34] Doring AC, Hageman MG, Mulder FJ (2014). Trigger finger: assessment of surgeon and patient preferences and priorities for decision making. J Hand Surg [Am].

[35] Durif-Bruckert C, Roux P, Morelle M, Mignotte H, Faure C, Moumjid-Ferdjaoui N (2015). Shared decision-making in medical encounters regarding breast cancer treatment: the contribution of methodological triangulation. Eur J Cancer Care (Engl).

[36] Gainer RA, Curran J, Buth KJ (2017). Toward optimal decision making among vulnerable patients referred for cardiac surgery: a qualitative analysis of patient and provider perspectives. Med Decis Mak.

[37] Ghane A, Huynh HP, Andrews SE (2014). The relative importance of patients' decisional control preferences and experiences. Psychol Health.

[38] Golden SE, Thomas CR, Moghanaki D (2017). Dumping the information bucket: a qualitative study of clinicians caring for patients with early stage non-small cell lung cancer. Patient Educ Couns.

[39] Gong HS, Huh JK, Lee JH (2011). Patients' preferred and retrospectively perceived levels of involvement during decision-making regarding carpal tunnel release. J Bone Joint Surg Am.

[40] Hack TF, Degner LF, Watson P (2006). Do patients benefit from participating in medical decision making? Longitudinal follow-up of women with breast cancer. Psychooncology..

[41] Hageman MG, Kinaci A, Ju K (2014). Carpal tunnel syndrome: assessment of surgeon and patient preferences and priorities for decision-making. J Hand Surg [Am].

[42] Hawley ST, Janz NK, Hamilton A (2008). Latina patient perspectives about informed treatment decision making for breast cancer. Patient Educ Couns.

[43] Heggland LH, Hausken K (2013). A qualitative identification of categories of patient participation in decision-making by health care professionals and patients during surgical treatment. Clin Nurs Res.

[44] Heggland LH, Hausken K (2014). Patient participation, decision-makers and information flow in surgical treatment. J Clin Nurs.

[45] Heggland LH, Mikkelsen A, Ogaard T (2014). Measuring patient participation in surgical treatment decision-making from healthcare professionals' perspective. J Clin Nurs.

[46] Henderson A, Shum D (2003). Decision-making preferences towards surgical intervention in a Hong Kong Chinese population. Int Nurs Rev.

[47] Henderson A, Shum D, Chien WT (2006). The development of picture cards and their use in ascertaining characteristics of Chinese surgical patients' decision-making preferences. Health Expect.

[48] Hopmans W, Damman OC, Senan S (2015). A patient perspective on shared decision making in stage I non-small cell lung cancer: a mixed methods study. BMC Cancer.

[49] Hou X-T, Pang D, Lu Q (2014). Preferred and actual participation roles in operation treatment decision making of patients with colorectal cancer. Int J Nurs Sci.

[50] Iaccarino JM, Simmons J, Gould MK (2017). Clinical equipoise and shared decision-making in pulmonary nodule management. A survey of American thoracic society clinicians. Ann Am Thorac Soc.

[51] Ihrig A, Keller M, Hartmann M (2011). Treatment decision-making in localized prostate cancer: why patients chose either radical prostatectomy or external beam radiation therapy. BJU Int.

[52] Janz NK, Wren PA, Copeland LA (2004). Patient-physician concordance: preferences, perceptions, and factors influencing the breast cancer surgical decision. J Clin Oncol.

[53] Johnson JD, Roberts CS, Cox CE (1996). Breast cancer patients' personality style, age, and treatment decision making. J Surg Oncol.

[54] Keating NL, Guadagnoli E, Landrum MB (2002). Treatment decision making in early-stage breast cancer: should surgeons match patients' desired level of involvement?. J Clin Oncol.

[55] Keating NL, Beth Landrum M, Arora NK (2010). Cancer patients' roles in treatment decisions: do characteristics of the decision influence roles?. J Clin Oncol.

[56] Lally RM (2009). In the moment: women speak about surgical treatment decision making days after a breast cancer diagnosis. Oncol Nurs Forum.

[57] Lam W, Fielding R, Chan M (2003). Participation and satisfaction with surgical treatment decision-making in breast cancer among Chinese women. Breast Cancer Res Treat.

[58] Lantz PM, Janz NK, Fagerlin A (2005). Satisfaction with surgery outcomes and the decision process in a population-based sample of women with breast cancer. Health Serv Res.

[59] Larsson US, Svardsudd K, Wedel H (1989). Patient involvement in decision-making in surgical and orthopaedic practice: the project perioperative risk. Soc Sci Med.

[60] Lee H, Lee YC, Shin S (2012). Participation and conflict in the decision-making process for endoscopic resection or surgical gastrectomy for early gastric cancer. J Surg Oncol.

[61] Markovic M, Manderson L, Quinn M (2006). Treatment decisions: a qualitative study with women with gynaecological cancer. Aust N Z J Obstet Gynaecol.

[62] Martinez KA, Resnicow K, Williams GC (2016). Does physician communication style impact patient report of decision quality for breast cancer treatment?. Patient Educ Couns.

[63] McGuire AL, McCullough LB, Weller SC (2005). Missed expectations? Physicians' views of patients' participation in medical decision-making. Med Care.

[64] Mendick N, Young B, Holcombe C (2010). The ethics of responsibility and ownership in decision-making about treatment for breast cancer: triangulation of consultation with patient and surgeon perspectives. Soc Sci Med.

[65] Meredith P (1993). Patient participation in decision-making and consent to treatment: the case of general surgery. Sociol Health Illn.

[66] Morgan JL, Burton M, Collins K (2015). The balance of clinician and patient input into treatment decision-making in older women with operable breast cancer. Psychooncology..

[67] Morishige R, Nakajima H, Yoshizawa K (2017). Preferences regarding shared decision-making in Japanese inflammatory bowel disease patients. Adv Ther.

[68] Moumjid N, Carrere MO, Charavel M (2003). Clinical issues in shared decision-making applied to breast cancer. Health Expect.

[69] Nam KP, Gong HS, Bae KJ (2014). The effect of patient involvement in surgical decision making for carpal tunnel release on patient-reported outcome. J Hand Surg [Am].

[70] Omar M, Tarplin S, Brown R (2016). Shared decision making: why do patients choose ureteroscopy?. Urolithiasis..

[71] Op den Dries S, Annema C, Berg AP (2014). Shared decision making in transplantation: How patients see their role in the decision process of accepting a donor liver. Liver Transpl.

[72] Orsino A, Cameron JI, Seidl M (2003). Medical decision-making and information needs in end-stage renal disease patients. Gen Hosp Psychiatry.

[73] Pieterse AH, Baas-Thijssen MC, Marijnen CA (2008). Clinician and cancer patient views on patient participation in treatment decision-making: a quantitative and qualitative exploration. Br J Cancer.

[74] Ramfelt E, Lutzen K, Nordstrom G (2005). Treatment decision-making in a group of patients with Colo-rectal cancer before surgery and a one-year follow-up. Eur J Cancer Care (Engl).

[75] Ratsep T, Abel A, Linnamagi U (2014). Patient involvement in surgical treatment decisions and satisfaction with the treatment results after lumbar intervertebral discectomy. Eur Spine J.

[76] Salkeld G, Solomon M, Short L (2004). A matter of trust--patient's views on decision-making in colorectal cancer. Health Expect.

[77] Santema TB, Stoffer EA, Kunneman M, et al. What are the decision-making preferences of patients in vascular surgery? A mixed-methods study BMJ Open 2017; 7: e013272. 10.1136/bmjopen-2016-013272 PMC5306515 28188153

[78] Seror V, Cortaredona S, Bouhnik AD (2013). Young breast cancer patients' involvement in treatment decisions: the major role played by decision-making about surgery. Psychooncology..

[79] Sidana A, Hernandez DJ, Feng Z (2012). Treatment decision-making for localized prostate cancer: what younger men choose and why. Prostate..

[80] Snijders HS, Kunneman M, Bonsing BA (2014). Preoperative risk information and patient involvement in surgical treatment for rectal and sigmoid cancer. Color Dis.

[81] Stiggelbout AM, Kiebert GM (1997). A role for the sick role. Patient preferences regarding information and participation in clinical decision-making. CMAJ..

[82] Sung VW, Raker CA, Myers DL (2010). Treatment decision-making and information-seeking preferences in women with pelvic floor disorders. Int Urogynecol J.

[83] Tyler Ellis C, Charlton ME, Stitzenberg KB (2016). Patient-reported roles, preferences, and expectations regarding treatment of stage i rectal cancer in the cancer care outcomes research and surveillance consortium. Dis Colon Rectum.

[84] Uldry E, Schafer M, Saadi A (2013). Patients' preferences on information and involvement in decision making for gastrointestinal surgery. World J Surg.

[85] Vogel BA, Helmes AW, Hasenburg A (2008). Concordance between patients' desired and actual decision-making roles in breast cancer care. Psychooncology..

[86] Wang AW, Chang SM, Chang CS (2018). Regret about surgical decisions among early-stage breast cancer patients: effects of the congruence between patients' preferred and actual decision-making roles. Psychooncology..

[87] Weiner BK, Essis FM (2006). Patient preferences regarding spine surgical decision making. Spine (Phila Pa 1976).

[88] Wilson A, Winner M, Yahanda A (2017). Factors associated with decisional regret among patients undergoing major thoracic and abdominal operations. Surgery..

[89] Woltz S, Krijnen P, Meylaerts SAG (2017). Shared decision making in the management of midshaft clavicular fractures: nonoperative treatment or plate fixation. Injury..

[90] Ziebland S, Evans J, McPherson A (2006). The choice is yours? How women with ovarian cancer make sense of treatment choices. Patient Educ Couns.

[91] Thom DH, Ribisl KM, Stewart AL, Luke DA (1999). Further validation and reliability testing of the Trust in Physician Scale. The Stanford trust study physicians. Med Care.

[92] Probst MA, Noseworthy PA, Brito JP (2018). Shared decision-making as the future of emergency cardiology. Can J Cardiol.

[93] Legare F, Ratte S, Gravel K (2008). Barriers and facilitators to implementing shared decision-making in clinical practice: update of a systematic review of health professionals' perceptions. Patient Educ Couns.

[94] Taylor LJ (2017). A framework to improve surgeon communication in high-stakes surgical decision best case/worst case. JAMA Surgery.

[95] Cai X, Robinson J, Muehlschlegel S (2015). Patient preferences and surrogate decision making in neuroscience intensive care units. Neurocrit Care.

