# Supplementary material for: Shared decision making in surgery: a scoping review of patient and surgeon preferences
Source: BMC Med Inform Decis Mak. 2020 Aug 12;20:190. doi: 10.1186/s12911-020-01211-0 (PMC7424662; doi:10.1186/s12911-020-01211-0)
Supplement: Supplementary file 1 — Additional file 1. Search strategies for Medline, Embase, and Cochrane databases. [file 12911_2020_1211_MOESM1_ESM.docx]

**Appendix A.** Search strategies for Medline, Embase, and Cochrane databases.

**Medline (n=10,665)**

1. *Decision Making/

2. *Negotiating/

3. *consensus/

4. *uncertainty/

5. *Choice Behavior/

6. exp *"Dissent and Disputes"/

7. Patient Preference/

8. "Physician-Patient Relations"/ and decision$.tw.

9. Patient Participation/ and decision$.tw.

10. ((shared or informed or directed or paternalistic or cooperative or collaborative or consensus) adj1 decision making).tw.

11. ((involved or involvement or uncertainty or participation) adj3 decision making).tw.

12. (decisional adj1 (conflict or regret or support or preparedness or preparation or assistance or needs)).tw.

13. (shared decision$1 or patient-directed decision$1 or physician-directed decision$1).tw.

14. (decision aid$1 or decision support tool$1).tw.

15. choice behavio?r$1.tw.

16. (patient preference$1 or patient choice$1).tw.

17. ((Patient empowerment or Patient participation or Patient engagement or Patient involvement) and decision$).tw.

18. 1 or 2 or 3 or 4 or 5 or 6 or 7 or 8 or 9 or 10 or 11 or 12 or 13 or 14 or 15 or 16 or 17

19. exp Surgical Procedures, Operative/

20. surgery.fs.

21. (surgery or surgeries or surgical or operation or operations or operative).tw.

22. 19 or 20 or 21

23. 18 and 22

24. (letter or comment or editorial).pt.

25. (ANIMALS not (HUMANS and ANIMALS)).hw.

26. (abortion or termination or pregnancy or infertility).tw.

27. 23 not (24 or 25 or 26)

**Embase (n=9,036)**

#36. #32 NOT (#33 OR #34 OR #35)

#35. [animals]/lim NOT [humans]/lim

#34. letter:it OR editorial:it

#33. abortion OR termination OR pregnancy OR infertility

#32. #31 AND english:la

#31. #22 AND #30

#30. #23 OR #24 OR #25 OR #26 OR #27 OR #28 OR #29

#29. surgical:ab,ti

#28. surgeries:ab,ti

#27. surgery:ab,ti

#26. 'operative procedure*':ab,ti

#25. 'surgical procedure*':ab,ti

#24. surgery:lnk

#23. 'surgery'/exp

#22. #1 OR #2 OR #3 OR #4 OR #5 OR #6 OR #7 OR #8 OR #9 OR #10 OR #11 OR #12 OR #13 OR #14 OR #15 OR #16 OR #17 OR #18 OR #19 OR #20 OR #21

#21. 'patient involvement':ab,ti AND decision*:ab,ti

#20. 'patient engagement':ab,ti AND decision*:ab,ti

#19. 'patient participation':ab,ti AND decision*:ab,ti

#18. 'patient empowerment':ab,ti AND decision*:ab,ti

#17. 'patient participation'/exp AND decision*:ab,ti

#16. 'doctor patient relation'/exp AND decision*:ab,ti

#15. 'patient choice*':ab,ti

#14. 'patient preference*':ab,ti

#13. 'patient preference'/exp

#12. 'choice behaviour*':ab,ti

#11. 'choice behavior*':ab,ti

#10. 'decision support tool*':ab,ti

#9. 'decision aid*':ab,ti

#8. 'physician-directed decision*':ab,ti

#7. 'patient directed decision*':ab,ti

#6. 'shared decision*':ab,ti

#5. (decisional NEAR/1 (conflict OR regret OR support OR preparedness OR preparation OR assistance OR needs)):ab,ti

#4. ((involved OR involvement OR uncertainty OR participation) NEAR/3 'decision making'):ab,ti

#3. ((shared OR informed OR directed OR paternalistic OR cooperative OR collaborative OR consensus) NEAR/1 'decision making'):ab,ti

#2. 'family decision making'/exp

#1. 'shared decision making'/exp

**Cochrane Library (n=658)**

#1 MeSH descriptor: [Decision Making] this term only

#2 MeSH descriptor: [Negotiating] this term only

#3 MeSH descriptor: [Dissent and Disputes] this term only

#4 MeSH descriptor: [Consensus] this term only

#5 MeSH descriptor: [Uncertainty] this term only

#6 ((shared or informed or directed or paternalistic or cooperative or collaborative or consensus) near/1 "decision making"):ti,ab

#7 ((involved or involvement or uncertainty or participation) near/3 "decision making"):ti,ab

#8 (decisional near/1 (conflict or regret or support or preparedness or preparation or assistance or needs)):ti,ab

#9 ("shared decision?" or "patient-directed decision?" or "physician-directed decision?"):ti,ab

#10 ("decision aid?" or "decision support tool?"):ti,ab

#11 MeSH descriptor: [Choice Behavior] this term only

#12 "choice behavio*r?":ti,ab

#13 MeSH descriptor: [Patient Preference] this term only

#14 "patient preference?":ti,ab or "patient choice?":ti,ab

#15 #1 or #2 or #3 or #4 or #5 or #6 or #7 or #8 or #9 or #10 or #11 or #12 or #13 or #14

#16 MeSH descriptor: [Physician-Patient Relations] this term only

#17 MeSH descriptor: [Patient Participation] this term only

#18 ("patient empowerment" or "patient participation" or "patient engagement" or "patient involvement"):ti,ab

#19 #16 or #17 or #18

#20 decision*:ti,ab

#21 #19 and #20

#22 #15 or #21

#23 MeSH descriptor: [Surgical Procedures, Operative] explode all trees

#24 Any MeSH descriptor with qualifier(s): [Surgery - SU]

#25 "surgical procedure?":ti,ab

#26 "operative procedure?":ti,ab

#27 (surgery or surgeries or surgical):ti,ab

#28 #23 or #24 or #25 or #26 or #27

#29 #22 and #28
